# 3D-assessment of RVOT dimensions prior percutaneous pulmonary valve implantation: comparison of contrast-enhanced magnetic resonance angiography versus 3D steady-state free precession sequence

**DOI:** 10.1007/s10554-019-01578-w

**Published:** 2019-04-01

**Authors:** Sebastian Ebel, Sebastian Gottschling, Maria T. A. Buzan, Matthias Grothoff, Ingo Dähnert, Robert Wagner, Daniel Gräfe, Philipp Lurz, Matthias Gutberlet, Christian Lücke

**Affiliations:** 1 Department of Diagnostic and Interventional Radiology, University of Leipzig – Heart Centre, Strümpellstrasse 39, 04289 Leipzig, Germany; 2 Department of Radiology, Addenbrooke’s Hospital, Cambridge University Hospitals NHS Foundation Trust, Cambridge, UK; 3 Department of Pediatric Cardiology and Congenital Heart Disease, University of Leipzig – Heart Centre, Leipzig, Germany; 4 Department of Pediatric Radiology, University of Leipzig, Leipzig, Germany; 5 Department of Cardiology, University of Leipzig – Heart Centre, Leipzig, Germany

**Keywords:** Tetralogy of Fallot, Magnetic resonance angiography, Pulmonary valve stenosis, Pulmonary valve Insufficiency, Preprocedural imaging

## Abstract

To compare contrast-enhanced magnetic resonance angiography (ceMRA) and 3D steady-state free precession (SSFP) during systole and diastole for assessment of the right ventricle outflow tract (RVOT) in patients considered for percutaneous pulmonary valve implantation (PPVI) after tetralogy of Fallot (TOF) repair. We retrospectively evaluated 89 patients (male: 45, mean age 19 ± 8 years), who underwent cardiac-MRI after surgical TOF-repair. Datasets covering the whole heart in systole and diastole were acquired using ECG-gated 3D SSFP and non-gated ceMRA. Measurements were performed in SSFP-sequences and in ceMRA in the narrowest region of the RVOT to obtain the minimum, maximum and effective diameter. Invasive balloon sizing as the gold standard was available in 12 patients. The minimum diameter in diastolic SSFP, systolic SSFP and ceMRA were 21.4 mm (± 6.1 mm), 22.6 mm (± 6.2 mm) and 22.6 mm (± 6.0 mm), respectively. Maximum diameter was 29.9 mm (± 9.5 mm), 30.0 mm (± 7.0 mm) and 28.8 mm (± 8.1 mm) respectively. The effective diameter was 23.2 mm (± 5.7 mm), 27.4 mm (± 6.7 mm) and 24.4 mm (± 6.2 mm), differing significantly between diastole and systole (p < 0.0001). Measurements in ECG-gated SSFP showed a better inter- and intraobserver variability compared to measurements in non-ECG-gated ceMRA. Comparing invasive balloon sizing with our analysis, we found the highest correlation coefficients for the maximum and effective diameter measured in systolic SSFP (R = 0.99 respectively). ECG-gated 3D SSFP enables the identification and characterization of a potential landing zone for PPVI. The maximum and effective systolic diameter allow precise sizing for PPVI. Patients with TOF-repair could benefit from cardiac MRI before PPVI.

## Introduction

Patients after surgical repair of tetralogy of Fallot (TOF) often suffer from pulmonary regurgitation (PR) or residual pulmonary stenosis (PS). The right ventricle (RV) generally tolerates PR well for years, but PS quickly can lead to RV-failure [1]. Often numerous surgeries with implantation and replacement of pulmonary conduits are required during a lifetime.

Althougn state-of-the-art-surgery of PI and PS in patients after TOF-repair has low mortality [2, 3], valve-carrying-conduits, however, have a limited lifespan of < 10 years so that the majority of the patients undergo multiple re-operations [4–7]. Percutaneous pulmonary valve implantation (PPVI) is a new treatment for PS and PR with excellent early and late results [8–10], it could help to delay surgery by prolonging conduit lifespan and reducing the number of operations on the open heart. It can be challenging to decide which patients are suitable for PPVI by using 2D-methods due to the complex 3D-anatomy of the right ventricle outflow tract (RVOT), especially after surgery. Furthermore, the occurrence of RVOT-aneurysms is a common complication after TOF-repair which may impede PPVI [11]. Since transcatheter-pulmonary-valves are available only in selected diameters [12], correct sizing of the RVOT in preparation to PPVI is crucial. Although non-invasive techniques are available, nowadays sizing for PPVI is still performed with invasive balloon-sizing [8, 13].

Two serious complications can occur during PPVI and the invasive preprocedural tests: occlusion/compression of the coronary vessels and perforation/rupture of the RVOT. Since PS requires dilation of the RVOT prior PPVI, perforation or rupture of the RVOT occurs more often in the condition of PS [14, 15]. Particularly in patients with congenital heart disease (CHD) after surgery, one cannot assume the coronary arteries to be “safely” away from the RVOT [8]. For risk-stratification of coronary-artery-compression, an invasive test was described which requires simultaneous inflation of a balloon catheter within the RVOT and injection of contrast medium through a second catheter placed in the aortic root [16]. If this test shows an occlusion of the coronaries by the balloon, PPVI should not be attempted. For risk-stratification of stent-/valve-fracture after PPVI the distance between the landing zone and the sternum is essential. Cardiac magnetic resonance imaging (CMR) can visualize the RVOT, its anatomy, and complications from TOF-repair [11]. Similar to computed tomography before transcatheter aortic valve replacement (TAVR), it might be possible to elucidate important features around the RVOT, the pulmonary valve and the coronaries with CMR, without using ionizing radiation [17].

In contrast to aortic root in TAVR, there is no defined landing zone inside the RVOT or pulmonary trunk (from now on referred to as RVOT) for PPVI. Thus it is necessary to identify the maximum systolic dimension of the narrowest diameter of the RVOT as a potential landing zone [9]. Simply measuring the diameter of the RVOT—generally oval-shaped—would result in inaccurate prosthesis-sizing. Since the effective diameter was introduced to optimize measurements [18], we will use this parameter for assessment of the RVOT in this study.

The purpose of our study is to determine which CMR-technique is better suited for the assessment of the RVOT in patients after TOF-repair considered for PPVI: breath-hold contrast-enhanced MR-angiography (ceMRA) or free-breathing navigator and ECG-gated 3D steady-state free precession (3D-SSFP) sequence, acquired during systole and diastole. Furthermore, we compared these techniques to invasive balloon sizing as the current gold standard. Additionally, we evaluate these techniques regarding reproducibility. Besides, we aim to assess the potential procedural complications, by measuring the distance from the potential landing zone to the left coronary artery (LCA) and for risk stratification of stent-fracture after PPVI the distance between from the landing zone to the sternum.

## Materials and methods

### Patient characteristics

Datasets of 89 consecutive patients after TOF-repair (45 males; mean age 19 ± 8 years) who underwent CMR as a follow-up after TOF-Repair between 06/2014 and 11/2015, were retrospectively reviewed. Most of the patients (n = 67) suffered from PR, a smaller group (n = 10) from a PS and the other participants (n = 12) from a combined impairment. Exclusion criteria were previous RVOT-stenting. All participants were free of contraindications for CMR. Informed consent for the use of the data was obtained from all subjects. Local ethics board approved the study. See Table 1 for details.

**Table 1:** Patient characteristics after surgical correction on Tetralogy of Fallot

Parameter	Total population (n = 89)	Pulmonary regurgitation^a^ (n = 67)	Pulmonary stenosis^b^ (n = 10)	Combined impairment^c^ (n = 12)
Pulmonary impairment	89 (100%)	67 (75.3%)	10 (11.2%)	12 (13.5%)
Age at CMR, median (range), SD	24.8 ± 12.7	23.8 ± 13.4	27.7 ± 9.9	28.4 ± 11.0
Age at surgical correction	2.3 ± 2.0	2.5 ± 2.2	1.7 ± 1.5	2.8 ± 2.1
Time passed since surgical correction	22.8 ± 11.0	21.6 ± 11.2	26.9 ± 9.5	25.7 ± 9.4
RVOT characteristics, n (%)				
Patchextended RVOT	83 (93%)	62 (74.7%)	10 (12%)	11 (13.3%)
Homograft	6 (7%)	5 (83%)	0 (0%)	1 (17%)
Underwent PPVI after MRI	12 (13%)	12 (100%)	0 (0%)	0 (0%)

^a^Regurgitant fraction of at least 30%

^b^RVOT gradient > 30 mmHg

^c^Patients who did not fit either the “predominantly pulmonary regurgitation” or “predominantry pulmonary stenosis”

### CMR image acquisition

All CMR studies were performed using a 1.5 T whole-body imaging system (Achieva, Philips Healthcare, Hamburg, Germany) together with a five-element phased-array body surface coil. Datasets, covering the whole heart were obtained using a free breathing navigator and ECG-gated 3D-steady state free precession sequence (for brevity we will further refer to the sequence as SSFP), and a contrast enhances MR-angiography. See Table 2 for details.

**Table 2:** Typical acquisition parameters of the different sequences

	TE (ms)	TR (ms)	FOV (mm)	Voxel size (mm)	Matrix (mm)	Slice thickness (mm)	Interslice GAP (mm)	Flip angle (°)	ECG-gating	Contrast agent
3D SSFP	2.3	4.7	300	1.25 × 1.25	240 × 256	2.74	1.37	90	Yes	None
ceMRA	1.14	2.5	480	1.40 × 1.40	424 × 372	3	1.5	30	None	15 mL Gadovist, followed by a bolus of 50 ml NaCl

*TE* time to echo, *TR* time to repeat, *FOV* Fielf’d of view, *ECG* electrocardiography

### CMR data analysis

#### Image quality

Image quality (IQ) of all datasets was assessed using a 5 point grading scale: *5-poor IQ*: not able to perform measurements; *4-impaired IQ*: barely able to perform needed measurements with limitations in defining large structures (i.e. distal segments of the pulmonary arteries); *3-intermediate IQ*: able to perform all measurements, but with limitations in defining medium-sized structures (i.e. proximal segments of the coronaries); *2-good IQ*: able to perform all measurements, with limitations in defining small structures (i.e. small or distal segments of the coronaries); *1-Excellent IQ*: no limitations.

#### RVOT dimensions

Measurements were performed using a dedicated postprocessing software (IntelliSpace Portal v8, Philips Healthcare systems, Best, Netherlands) by two radiologists, each with > 4 years of experience in post-processing.

**Fig. 1:**
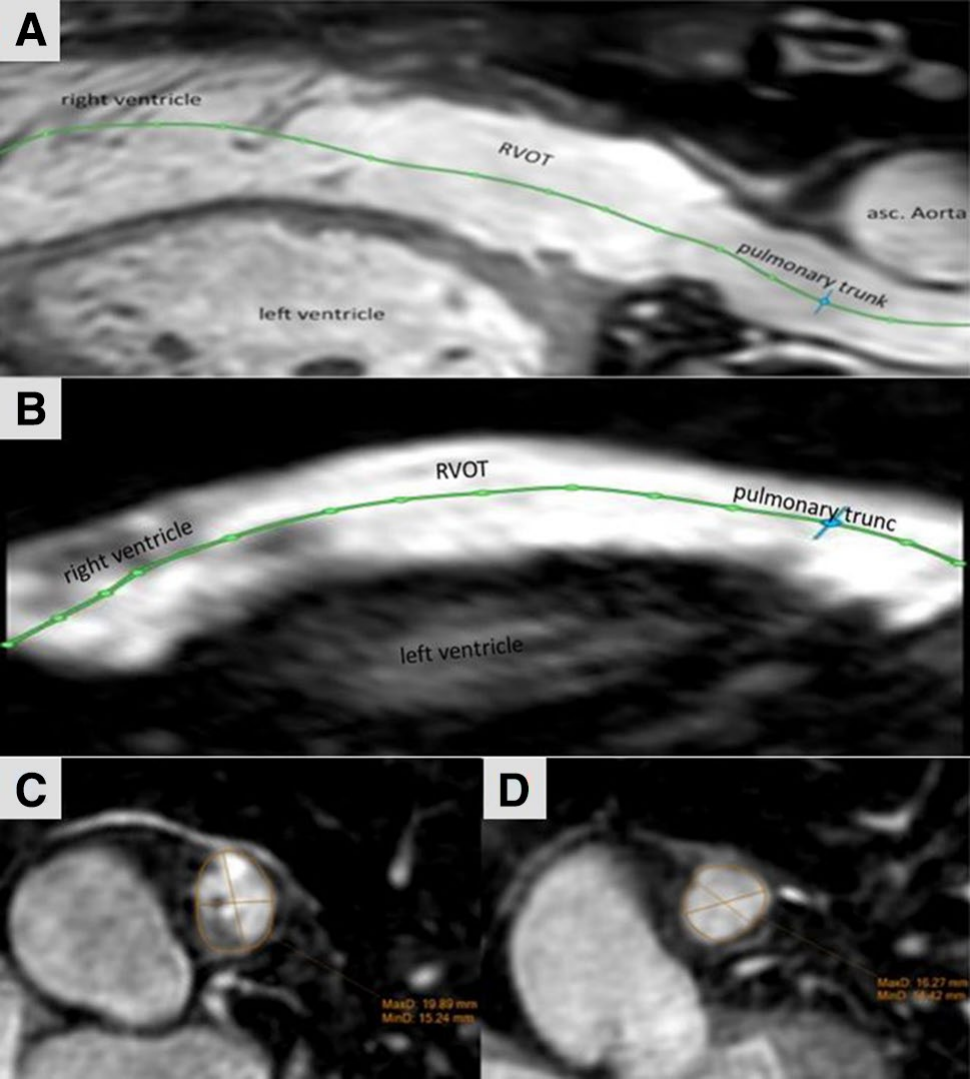
**a, b** Curved multiplanar reformats of the right ventricle, the right ventricle outflow tract (RVOT) and the pulmonary trunk reconstructed from the 3D steady-state free precession (SSFP) (**a**) “whole heart” images during systole and from ceMRA (**b**). The centerline (green) starts at the bottom of the right ventricle, proceeds through the RVOT and the pulmonary trunk and ends at the pulmonary bifurcation. All measurements were carried out perpendicular to the centerline. **c, d** Multiplanar reformations of a cross-section of the pulmonary trunk in SSFP-sequences showing the change in shape and size at the exact same level during systole and diastole

The RVOT was assessed by defining a centerline starting at the bottom (in axial slices) of the right ventricle and ending at the pulmonary bifurcation (Fig. 1). All measurements were carried out perpendicular to this centerline. The RVOT was defined as the area between the crest of the right ventricle and the pulmonary valve [19]. The area without any trabeculation below the pulmonary valve defined the crest of the RV. The effective diameter was defined as the diameter of an idealized circle with the area of the evaluated region. We measured the minimum and maximum diameters of the RVOT in SSFP sequences during systole and diastole and in ceMRA [20]. Manual feature tracking and the distance to the pulmonary bifurcation were used as reliable landmarks to guarantee measurements at exact the same position in all sequences. The effective diameter was calculated as previously described using the following formula [21]:

$${\text{EDarea }}={\text{ 2}} \times \surd \left( {{\text{area}}/\pi } \right)$$

The area was assessed by drawing a region of interest (ROI) around the perimeter of the RVOT.

**Fig. 2:**
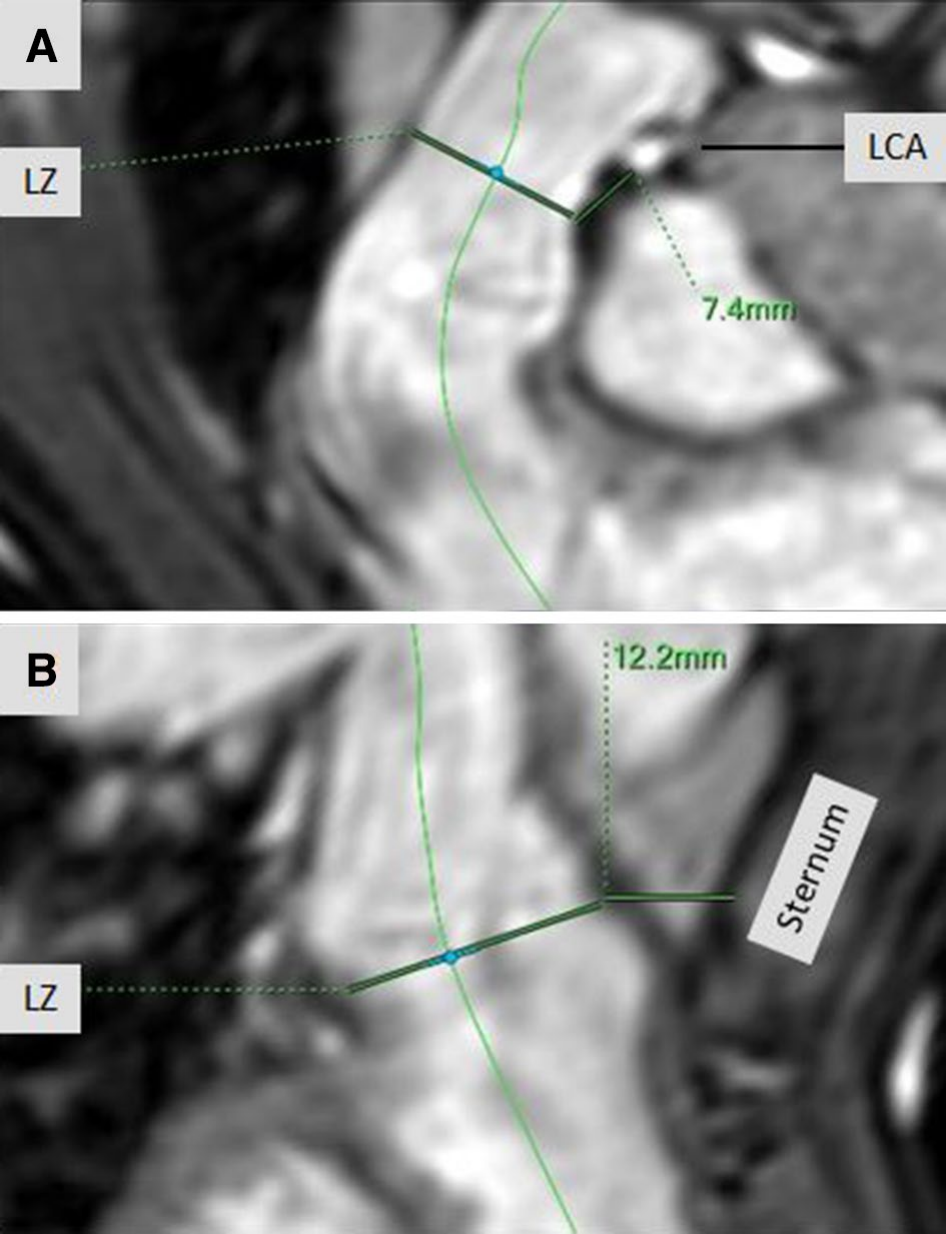
Curved multiplanar reformats of the right ventricle outflow tract and the pulmonary trunk reconstructed from the 3D steady-state free precession (SSFP) “whole heart” images during systole. **a** Shows the measurements of the distance between the possible landing zone (landing zone) and the left coronary artery (LCA). **b** Shows the measurements of the distance between the possible landing zone (landing zone) and the sternum

Additionally, we measured the shortest distances between the potential landing zone and the LCA and between the landing zone and the inner surface of the sternum (Fig. 2).

### Intra- and Interobserver variability

The intraobserver variance was assessed by one investigator with > 4 years of experience in post-processing. This observer performed the previously described measurements in all 89 datasets and repeated them in random order 4 weeks after the first evaluation. The interobserver variance was analyzed in a subgroup of 35 randomly selected datasets by the second investigator, according to the above-described methodology.

### cMRI-measurements versus invasive measurements (gold standard)

In a subgroup of 12 participants who underwent PPVI after cMRI, we compared our measurements with invasive balloon sizing. Correlation coefficients and Bland-Altman-Analysis was performed.

### Statistical analysis

Statistical analysis was performed with MedCalc Statistical Software v15.11.4. (MedCalc Software, Ostend, Belgium). Qualitative data were expressed as absolute values and percentages. Quantitative variables were expressed as mean values and standard deviations. To check for normal distribution of the data, a Shapiro-Wilks test was performed. Once normality was proven a paired t-test was performed. A p-value < 0.05 was considered statistically significant. Correlation analyses were performed using the Pearson rank correlation. Intra- and interobserver variability was assessed using linear regression analysis, scatter-, Bland-Altman-plots and interclass correlation (ICC). Bland-Altman-analysis, providing the mean differences between measurements (bias), the standard deviation and the limits of agreement (LOA), was used for the different measurements.

## Results

### Image quality

The mean overall IQ grading of all sequences was 2.4 ± 0.7. Datasets of five (5.6%) very anxious patients were not considered due to poor image quality (grade 5) related to severe motion artifacts. The SSFP sequences showed a mean image quality of 2.2 ± 0.7 during systole and 2.3 ± 0.7 during diastole, indicating that the majority of patients had a rather good or excellent IQ. In ceMRA the mean IQ was 2.8 ± 0.6, indicating that most of the patients had only an intermediate to good IQ. The differences in IQ between ceMRA and SSFP were statistically significant (p < 0.05), but not the differences of IQ between systole and diastole in SSFP (p = 0.2). Regarding image quality we found no differences between patients with PR and patients with PS (p = 0.3). The distribution of IQ-scores is shown in Table 3.

**Table 3:** Distribution of the image quality of all datasets

Grade	ceMRA	SSFP systole	SSFP diastole
	N (%)	N (%)	N (%)
5—Poor	5	5	5
	4.45%	4.45%	4.45%
4—Impaired	7	5	5
	6.23%	4.45%	4.45%
3—Intermediate	53	22	17
	47.17%	19.58%	15.13%
2—Good	24	58	54
	21.36%	51.62%	48.06%
1—Excellent	0	10	8
	0%	8.9%	7.12%

Grade 5: No measurements are possible

Grade 4: Measuring is barely possible

Grade 3: Measuring is possible with limitation in definition of medium sized structures

Grade 2: Limitations in definition of small-sized structures

Grade 1: No limitation at all

*ceMRA* contrast-enhanced magnetic resonance angiography, *SSFP systole* 3D-steady state free precession during systole, *SSFP diastole* 3D-steady state free precession during diastole

### RVOT dimensions

We found a wide range in width of the RVOT. The smallest potential landing zone in systole had a maximum diameter of 22.1 mm, the largest 46.2 mm. We observed that the shape of the landing zone changes considerably during the cardiac cycle. Shown in Fig. 1.

#### Effective Diameter

**Fig. 3:**
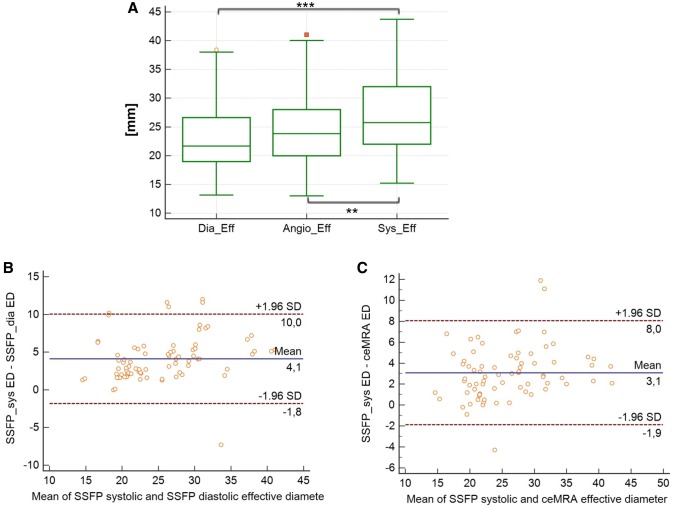
**a** Box-plot of the mean effective diameter in SSFPdiastole, ceMRA, and SSFPsystole. Triple asterisk indicate significant differences between SSFPdiastole and SSFPsystole (p < 0.001). **b** Bland Altman analysis of the mean effective diameter, measured in SSFP systole and SSFP diastole. The mean effective diameter in systole is measured 4.1 mm larger than the mean effective diameter in diastole. **c** Bland Altman analysis of the mean effective diameter, measured in SSFP systole and ceMRA. The mean effective diameter in systole is measured 3.1 mm larger than the mean effective diameter in ceMRA. *ED*_*sys*_ Effective diameter, measured in SSFP acquired during systole, *ED*_*dia*_ Effective diameter, measured in SSFP acquired during diastole, *ED*_*ceMRA*_ Effective diameter, measured in ceMRA

**Fig. 4:**
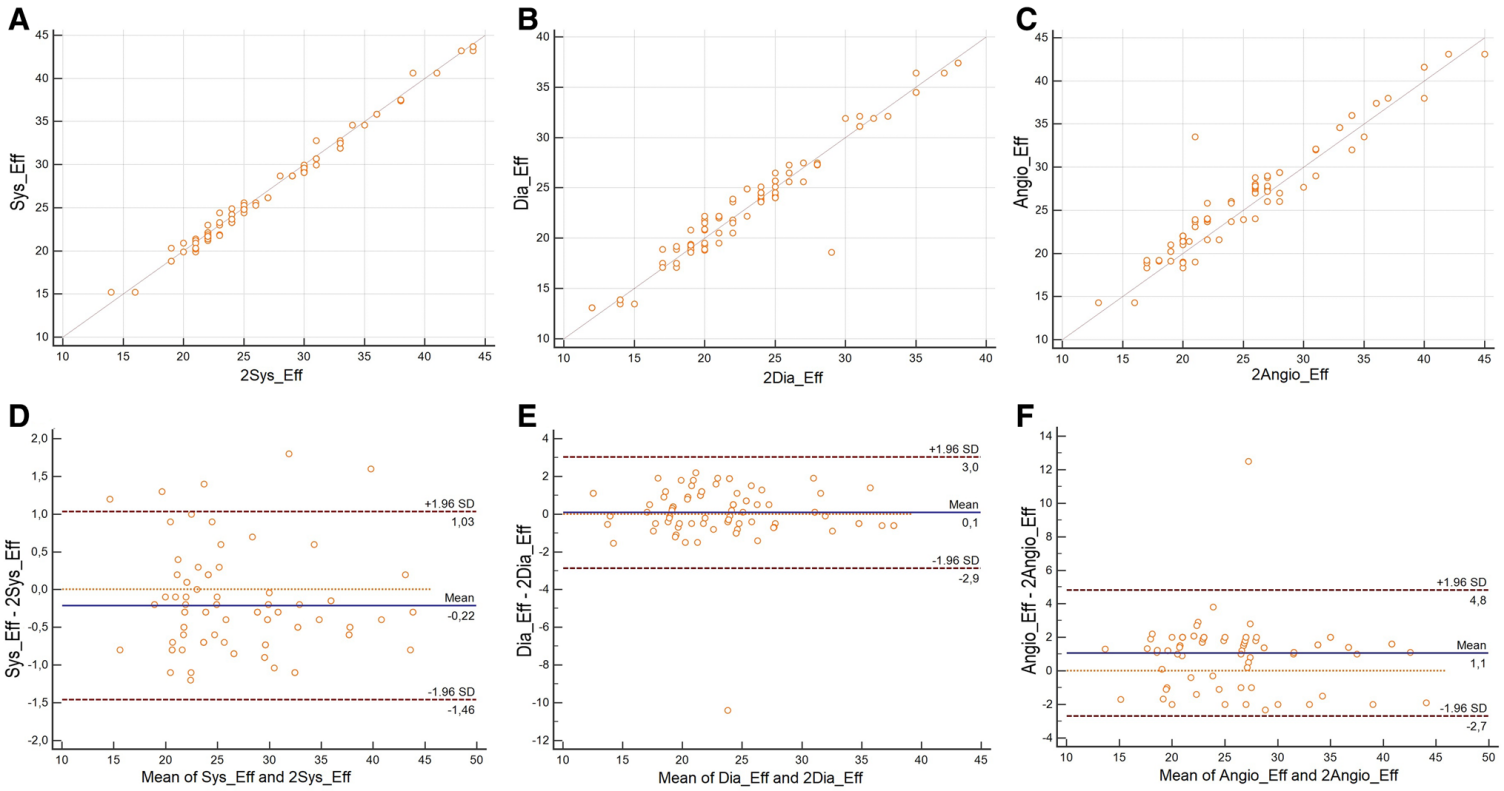
Scatterplot of the interobserver variability of measurements of the effective diameter of a possible landing zone in SSFP systole **(a)** (Correlation coefficient (R) = 0.8466; p < 0.0001), SSFP diastole **(b)** (r = 0.8348; p < 0.0001) and ceRMA **(c)** (r = 0.5507; p = 0.0007). Bland Altman-analysis of interobserver variability **(d–f)**

The mean effective diameter of a potential landing zone was significantly larger during SSFP-systole 27.4 mm (± 6.7 mm) as compared to SSFP-diastole 23.2 mm (± 5.7 mm) and ceMRA images 24.4 mm (± 6.2 mm), p < 0.001, with a standard error of the mean (SEM) between 0.38 and 0.43 mm. See Figs. 3 and 4 for details.

Mean differences of the effective diameters as measured during systole and diastole on SSFP were 4.1 mm and 3.1 mm, respectively, when compared to measurements on ceMRA. The LOA are presented in Fig. 4a–c. The best agreement was achieved with measurements in SSFP-systole. We found no differences between patients with PR and PS (p = 0.2).

#### Minimum and Maximum Diameter

The mean minimum diameter in SSFP-systole was 22.6 mm (± 6.2 mm), slightly smaller in SSFP-diastole with 21.4 mm (± 6.1 mm), but similar in ceMRA with 22.6 mm (± 6.0 mm), with no significant statistical differences. The mean maximum diameter in SSFP-systole was 30.0 mm (± 7.0 mm), in SSFP-diastole 29.9 mm (± 9.5 mm) and in ceMRA 28.8 mm (± 8.1 mm), with no significant differences between measurements (Table 4).

**Table 4:** Measurements

	SSFPsystole (mm)	SSFPdiastole (mm)	ceMRA (mm)	p-value/SEM SSFPsystole versus SSFPdiastole (mm)	p-value/SEM SSFPsystole versus ceMRA (mm)	p-value/SEM SSFPdiastole versus ceMRA (mm)	LOA SSFPsystole versus SSFPdiastole (mm)	LOA SSFPsystole versus ceMRA (mm)
Minimal diameter	22.6 ± 6.1	21.4 ± 6.1	22.6 ± 6.0	0.07/0.65	0.79/0.6	0.053/0.24	− 7.5 to + 9.9	− 6.6 to + 8.8
Maximal diameter	30.0 ± 7.0	29.9 ± 9.5	28.8 ± 8.1	0.947/1.09	0.14/0.83	0.4043/1.08	− 15.1 to + 15.2	− 10.4 to + 12.4
Effective diameter	27.4 ± 3.1	23.2 ± 5.7	24.4 ± 6.2	< 0.0001/0.43	< 0.0001/0.42	0.0017/0.38	− 1.8 to + 10.0	− 1.9 to + 8.0

Mean minimum, maximum and effective diameter in SSFP diastole, SSFP systole and ceMRA with standard deviation, p-values, and LOA. Student’s t-test was performed to obtain p-values

*SSFPsystole* 3D-steady state free precession during systole, *SSFPdiastole* 3D-steady state free precession during diastole, *ceMRA* contrast enhanced magnetic resonance angiography, *SEM* standard error of the mean, *LOA* Limits of agreement

#### Intraobserver variability

Analysis of the intraobserver variability showed for the measurements of the effective diameter on SSFP sequences R = 0.95 (p < 0.0001), ICC 0.79 when measured during systole and R = 0.90 (p < 0.0001), ICC 0.76 during diastole. Even in ungated ceMRA sequences a correlation coefficient of R = 0.74 (p < 0.01), ICC 0.72 could be achieved. (Fig. 4a–c).

Bland–Altman analysis demonstrated a mean difference of the effective diameter as measured during SSFP-systole of − 0.22 mm and of 0.1 mm when measured in SSFP-diastole and of 1.1 mm when measured in ceMRA, indicating a small intraobserver variability on SSFP-images and ceMRA images. LAO were: SSFPsystolic − 1.46 to + 1.03 mm, SSFPdiastolic − 2.9 to + 3.0 mm and ceMRA − 2.7 to + 4.8 mm. (Fig. 4d, e).

#### Interobserver variability

**Fig. 5:**
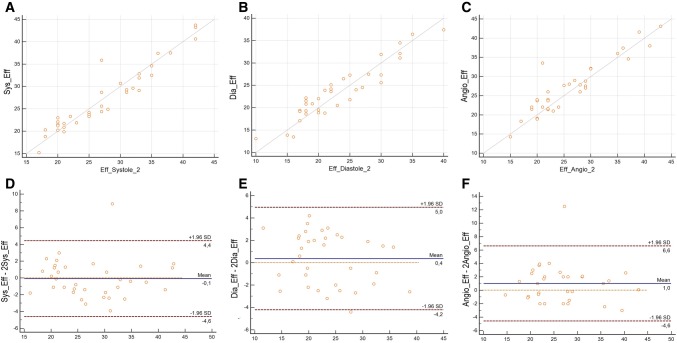
Scatterplot of the intraobserver variabilty of measurements of the effective diameter of a possible landing zone in SSFP systole **(a)** (Correlation coefficient (R) = 0.9467; p < 0.0001), SSFP diastole **(b)** (r = 0.9043; p < 0.0001) and ceRMA **(c)** (R) = 0.7336; p = 0.0028. Bland Altman analysis of interobserver variability **(d–f)**

The lowest interobserver variabilities could be achieved when measuring the effective diameter on SSFP sequences with R = 0.85 (p < 0.0001) for the measurements of the effective diameter during systole and R = 0.84 (p < 0.0001) during diastole (Fig. 5a–c), whereas measurements in ceMRA demonstrated only R = 0.55 (p < 0.001).

Bland–Altman analysis demonstrated a mean difference of the effective diameter as measured during SSFP-systole of -0.1 mm and of 0.4 mm when measured during diastole and of 1.0 mm when measured in ceMRA. This indicates a lower interobserver variability on SSFP-images during systole as compared to diastole or on ceMRA. LOA were: SSFPsystolic − 4.6 to + 4.4 mm, SSFPdiastolic − 4.2 to + 5.0 mm and ceMRA − 4.6 to + 6.6 mm (Fig. 5d, e).

The interobserver variability for maximum diameter measurements was higher for all sequences with R = 0.47 (p < 0.01) when measured with SSFP during systole, R = 0.57 (p < 0.001) for SSFP during diastole and R = 0.57 (p < 0.001) when ceMRA was used.

#### Validation of cMRI-measurements against a gold-standard

**Fig. 6:**
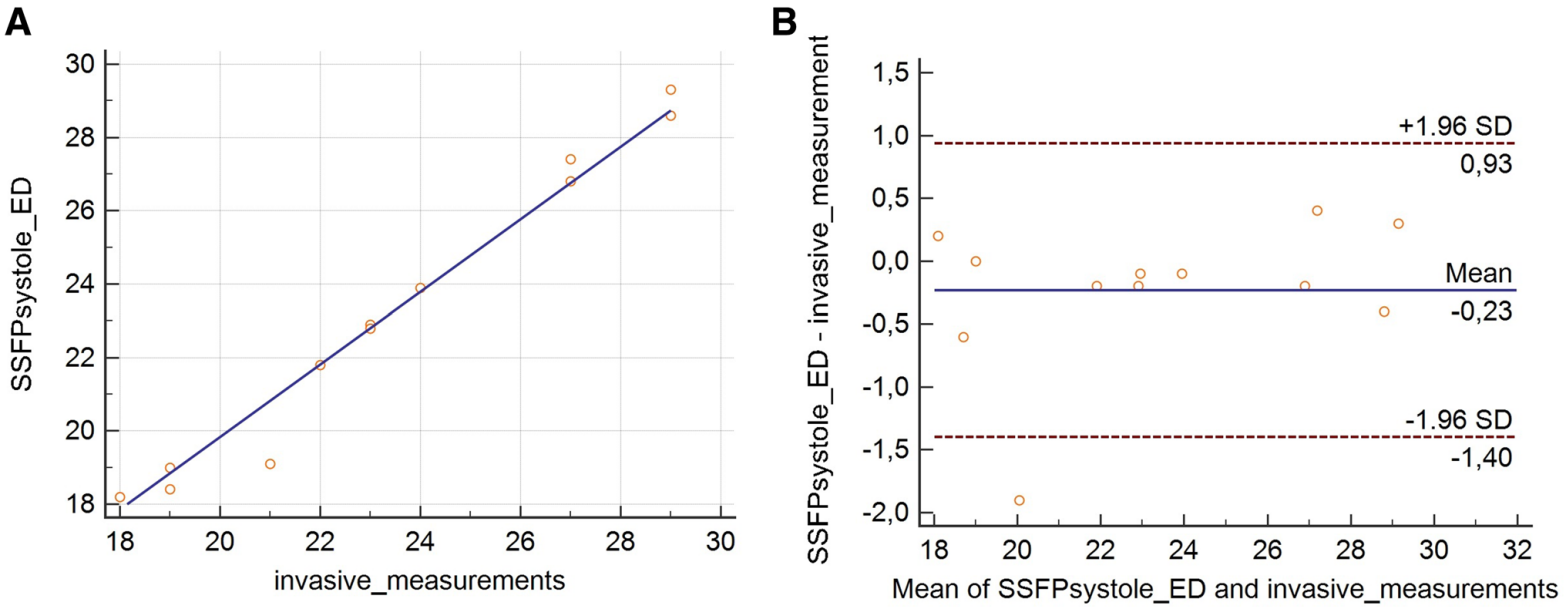
Scatterplot of comparison of measurements of the effective diameter of a possible landing zone in SSFP systole and invasive measurements **(a)** (Correlation coefficient (R) = 0.99; p < 0.0001) **(b)** Bland Altman analysis showed no relevant over- or undersizing and narrow limits of agreement

Comparing invasive balloon sizing with our measurements we found the highest correlation coefficients (R = 0.99 respectively) for the maximum and effective diameter measured in SSFPsystole. The other results are shown in Table 5. Bland–Altman analysis demonstrated a bias in sizing as measured during systole when compared to invasive measurements of − 0.23 mm. LAO were: 0.93 to − 1.4 mm (Fig. 6a, b).

**Table 5:** Comparison of invasive and CMR measurements

	Correlation coefficient r versus Balloonsizing	p-value versus Balloonsizing
SSFPsystole minimal diameter	0.88	< 0.05
SSFPsystole maximal diameter	0.99	< 0.0001
SSFPsystole effective diameter	0.99	< 0.0001
SSFPdiastole minimal diameter	0.86	0.07
SSFPdiastole maximal diameter	0.85	0.06
SSFPdiastole effective diameter	0.85	0.06
ceMRA minimal diameter	0.72	0.2
ceMRA maximal diameter	0.70	0.2
ceMRA effective diameter	0.55	0.3

*SSFPsystole* 3D-steady state free precession during systole, *SSFPdiastole* 3D-steady state free precession during diastole, *ceMRA* contrast-enhanced magnetic resonance angiography, *SEM* standard error of the mean, *LOA* Limits of agreement

#### Preprocedural risk

In each case, the course of the LCA was near the RVOT. In ceMRA it was not possible to evaluate the coronary vessels due to the early timing for peak enhancement in the pulmonary circulation as well as due to motion artifacts in the absence of ECG gating. In ECG-synchronized SSFP-sequences it was possible to visualize the proximal LCA in all of our patients. We found no significant differences between measurements during systole or diastole (p = 0.30).

Regarding the shortest distance between the landing zone for PPVI and the inner surface of the sternum, we found no significant differences in measurements between SSFP-systole and SSFP-diastole (p = 0.80), between SSFP-systole and ceMRA (p = 0.084) and between SSFP-diastole and ceMRA (p = 0.72).

## Discussion

This study shows that CMR is a suitable technique for preprocedural assessment of the RVOT and for sizing before PPVI in patients after TOF repair. Measurements in systolic SSFP showed excellent correlation with the current gold standard and are therefore suitable for precise prosthesis sizing. We found a better performance of 3D-whole-heart-SSFP against ceMRA regarding reproducibility and image quality. Additionally, we showed that it is mandatory to use ECG-gated sequences.

We found better image quality in SSFP sequences compared to ceMRA. Additionally, SSFP sequences enabled evaluation of the pulmonary, systemic and venous vasculature in one acquisition while ceMRA depends strongly on timing for contrast media and therefore must be performed several times to enable evaluation of all compartments of the vasculature. Patients with CHD receive several follow-up CMR-examination during their lifetime, and since there is a risk of tissue damage, extravasation or gadolinium-accumulation in the brain when using contrast-medium, imaging techniques without the need for contrast agent, like SSFP-sequences, should be preferred [22].

Our study verifies that the diameter of the RVOT depends strongly on the timing of acquisition within the cardiac phase: wider in SSFP-systole, smaller in SSFP-diastole and in between in ceMRA without ECG-gating. Significant differences regarding the effective diameter of the RVOT between ECG-gated SSFP-sequences and non-gated ceMRA occur, suggesting that single measurements in not-ECG-gated sequences could lead to underestimating the true maximum diameter of the RVOT. These findings agree with the literature, that contrast-enhanced 4D computed tomography is suitable for obtaining information about the dynamics of RVOT during the cardiac cycle [23]. However, there are general limitations of CT, which must be considered—especially in young patients: high radiation-dose in young patients, need of potentially nephrotoxic contrast medium and limited assessment of the pulmonary valve/conduit function. Measurements in 2D-datasets from invasive measurements or echocardiography can be inaccurate because of “misangulation” and shifting of the potential landing zone out-of-plane during the cardiac cycle. This underlines the role of preprocedural imaging and the importance for ECG-gated 3D-datasets for accurate measurements.

We showed that the cross-sections of a potential landing zone are mostly oval-shaped rather than circular structures. Simple measurements of the minimum or maximum diameter would lead to over- or underestimation of its true size, so it seems reasonable to consider the effective diameter like it is done for TAVR-planning [17]. Measurements of the effective diameter in SSFP-sequences showed better inter- and intraobserver variability than measurements in ceMRA. Since patients after TOF-repair receive frequent follow-up examinations it is mandatory to use sequences with the best intra- and interobserver variability to obtain consistent data. These findings fit with literature, showing a good reproducibility of measurements of the RV-dimensions preformed in SSFP sequences in patients with CHD [24].

Comparing MRI measurements with invasive measurements as a gold standard, we found an excellent correlation for the maximum and effective diameter in SSFPsystole. Which underlines our hypothesis the effective diameter in systole is a reliable parameter to predict the needed valve size for PPVI. Since noninvasive preprocedural MRI is suitable for precise prosthesis sizing it could help in reducing radiation dose and contrast volume during needed during the procedure of PPVI.

Due to the risk of compression/occlusion of the LCA during PPVI, the assessment of the coronaries is essential [8, 18, 25]. Hence it was not possible to assess the coronaries is ceMRA the authors conclude that simple single-phase, non-ECG-gated MR-angiography is not suitable for assessment of the RVOT before PPVI. The authors recommend assessment of the coronaries non-invasively in ECG-gated SSFP sequences, preferably in systole. Since there are no guidelines what should be the minimum distance that would prevent occlusion of the coronaries, there is a need for further comparison between invasive tests and CMR, so that it might be possible to spare invasive testing in favor of CMR in the future. Additionally, there are no guidelines what would be an optimal distance between stent and sternum. This issue should be addressed in further studies.

One limitation of this study is that first pass ceMRA is an imaging technique acquired in breath-hold, but without ECG-gating. This is the standard protocol and we could not control for that due to the retrospective nature of the study. An ECG-gated first pass ceMRA is not available.

In conclusion, 3D-SSFP is feasible for the evaluation of a potential landing zone for PPVI. We recommend 3D-SSFP for preprocedural assessment of patients after surgical TOF-repair who could benefit from PPVI. Obtaining ECG-gated sequences for proper and reliable measurements is mandatory. We showed that high-resolution SSFP sequences acquired during systole allow better evaluation of a potential landing zone for PPVI regarding its morphology and its relation to the left coronary vessel and sternum. The maximum and effective systolic diameters show good correlation with invasive measurements and allow precise sizing for PPVI without the need for contrast agent.
